# Prevention of nosocomial COVID-19: Another challenge of the pandemic

**DOI:** 10.1017/ice.2020.166

**Published:** 2020-04-23

**Authors:** Jens T. Van Praet, Bram Claeys, Ann-Sofie Coene, Katelijne Floré, Marijke Reynders

**Affiliations:** 1 Department of Nephrology and Infectious Diseases, AZ Sint-Jan Brugge-Oostende AV, Brugge, Belgium; 2 Department of Internal Medicine and Pediatrics, Ghent University, Ghent, Belgium; 3 Faculty of Medicine and Health Sciences, Ghent University, Ghent, Belgium; 4 Faculty of Medicine, KU Leuven, Leuven, Belgium; 5 Department of Laboratory Medicine, Medical Microbiology, AZ Sint-Jan Brugge-Oostende AV, Brugge, Belgium

*To the Editor*—Coronavirus disease 2019 (COVID-19) is an illness caused by severe acute respiratory syndrome coronavirus 2 (SARS-CoV-2), a recently emerged novel virus that is currently spreading globally.^1^ Its clinical presentation varies from mild, sometimes unrecognized, respiratory symptoms to overwhelming pneumonia and multiple-organ failure leading to death. The virus is probably mainly transmitted via respiratory droplets and can survive on surfaces for several days. The incubation period varies between 4 and 14 days, and patients can be contagious before the onset of symptoms.^2^ The duration of infectivity is uncertain, with one study reporting that 90% of mild cases had a negative real-time polymerase chain reaction (PCR) test by day 10.^3^

On March 13, 2020, the first outpatient with COVID-19 was admitted to our hospital, a 1,182-bed acute- and tertiary-care hospital in Belgium consisting of 3 separate campuses. In week 13 (March 16–22), we observed that 4 patients who had been hospitalized for other reasons presented with COVID-19 (Table 1). COVID-19 was diagnosed based on a positive real-time PCR test from a nasopharyngeal swab and/or the presence of typical radiographic abnormalities on a computed tomography (CT) scan of the lungs. Because the hospitalization duration of these patients clearly exceeded the minimal incubation period, these infections were considered nosocomially acquired, transmitted by healthcare workers or external visitors. We implemented several measures to prevent further cases of nosocomial transmission. First, from the beginning of week 12, we screened all healthcare personnel with direct patient contact for cases of low-grade fever (>37.4°C) and acute developing or worsening respiratory symptoms and tested possible cases using nasopharyngeal swabs and real-time PCR. Positive screening resulted in removal from the work floor for 14 days.

**Table 1.** Characteristics of the Nosocomial COVID-19 Cases in Week 13

Patient Sex and Age	Days of Hospitalization Before Onset Symptoms	Original Reason for Admission	Clinical Symptoms of COVID-19	Outcome
Female, 78 y	40	Humerus fracture	Fever (>38.5°C) and cough	Full recovery
Male, 75 y	25	Acute kidney injury and nephrolithiasis	Fever (> 38.5°C)	Full recovery
Female, 90 y	32	Lumbalgia	Fever (>38.5°C) and cough	Full recovery
Male, 65 y	8	Cholangitis with underlying liver metastases	Low-grade fever (>38.2°C) and respiratory failure	Deceased

Furthermore, we cancelled all elective consultations and procedures, and we prohibited visits to the hospital with restrictive exceptions for intensive care, pediatric wards, and obstetric wards, as required by government regulations from March 13 (end of week 12). From this day forward, all healthcare workers were obligated to wear surgical masks as personal protective equipment during patient contact, regardless of their own symptoms. Additionally, we created physically separated wards for patients with or without COVID-19.

The number of admissions of outpatients with COVID-19 increased from 5 in week 12 to 22 in week 13, 49 in week 14 and 42 in week 15, illustrating the increasing incidence of COVID-19 during the beginning of the epidemic in Belgium. In these same weeks, the screening positivity rates of symptomatic healthcare workers in our hospital were 8.6% (6 out of 70), 31% (17 out of 54), 39% (16 out of 41) and 28% (16 out of 57), respectively and the numbers of patients diagnosed with probable nosocomial COVID-19 were 0, 4, 4, and 23, respectively. We defined probable nosocomial COVID-19 as a diagnosis made beyond 4 days of hospitalization and the absence of clinical suspicion of COVID-19 upon admission. Of 31 probable nosocomial COVID-19 infections, 22 (71%) were observed at geriatric wards.

Our data indicate that nosocomially acquired COVID-19 can be observed at the start of a local epidemic and represents another challenge of the pandemic. Despite diverse and strictly followed preventive measures, we observed increasing numbers of new cases in our hospital during the first weeks of the epidemic, especially in geriatric wards. Further studies are required to identify the optimal preventive approach, which will probably include the regular screening of all asymptomatic healthcare personnel working at wards with high rates of nosocomial transmission.
